# Analysis of the competitiveness between a non-aflatoxigenic and an aflatoxigenic *Aspergillus flavus* strain on maize kernels by droplet digital PCR

**DOI:** 10.1007/s12550-021-00447-7

**Published:** 2021-12-15

**Authors:** Alexandra Schamann, Markus Schmidt-Heydt, Rolf Geisen

**Affiliations:** Department of Safety and Quality of Fruit and Vegetables, Max Rubner-Institut (MRI) - Federal Research Institute of Nutrition and Food, Karlsruhe, Germany

**Keywords:** ddPCR, Non-aflatoxigenic *Aspergillus flavus*, Biocontrol, Aflatoxin, Maize, Molecular monitoring

## Abstract

Non-aflatoxigenic *Aspergillus flavus* strains are used as a biocontrol system on maize fields to decrease the aflatoxin biosynthesis of aflatoxigenic *A. flavus* strains. *A. flavus* strain AF36 was the first commercially available biocontrol strain and is authorized for use on maize fields by the US Environmental Protection Agency, e.g., in Texas and Arizona. A droplet digital PCR (ddPCR) assay was developed to analyze the mechanisms of competition and interaction of aflatoxigenic and non-aflatoxigenic *A. flavus* strains. This assay enables the parallel identification and quantification of the biocontrol strain *A. flavus* AF36 and the aflatoxigenic *A. flavus* strain MRI19. To test the assay, spores of both strains were mixed in varying ratios and were incubated on maize-based agar or maize kernels for up to 20 days. Genomic equivalent ratios (genome copy numbers) of both strains were determined by ddPCR at certain times after incubation and were compared to the spore ratios used for inoculation. The aflatoxin biosynthesis was also measured. In general, *A. flavus* MRI19 had higher competitiveness in the tested habitats compared to the non-aflatoxigenic strain, as indicated by higher final genomic equivalent ratios of this strain compared to the spore ratios used for inoculation. Nevertheless, *A. flavus* AF36 effectively controlled aflatoxin biosynthesis of *A. flavus* MRI19, as a clear aflatoxin inhibition was already seen by the inoculation of 10% spores of the biocontrol strain mixed with 90% spores of the aflatoxigenic strain compared to samples inoculated with only spores of the aflatoxigenic *A. flavus* MRI19.

## Introduction

*Aspergillus flavus* is a saprophytic and weakly pathogenic fungus of plants, which infects many economically relevant crops such as cereals, nuts, and many other plant commodities (Calderari et al. 2013; Andrade and Caldas 2015; Wu et al. 2016). Especially in countries with a warm climate, certain crops are prone to infections with *A. flavus*, which can occur at the pre- and postharvest stages, leading to contamination with aflatoxins (AFs), which are toxic secondary metabolites of the fungus (Samuel et al. 2013). Toxigenic strains of *A. flavus* synthesize aflatoxin B_1_ (AFB_1_) and B_2_, whereas other *Aspergillus* species like *A. parasiticus* additionally synthesize aflatoxin G_1_ and G_2_ (Frisvad et al. 2019). AFB_1_ has been classified as a group 1 carcinogen for humans by the International Agency for Research on Cancer (IARC 2012).

For several decades, various approaches have been tested to reduce aflatoxin (AF) contamination in food and animal feed. At pre-harvest stages, the development of resistant maize lines by traditional and molecular breeding methods and the application of microorganisms such as yeast (e.g., *Wickerhamomyces anomalus*) or bacteria (e.g., *Bacillus subtilis*) as biocontrol systems have been intensively studied (Cary et al. 2011; Shifa et al. 2016; Hua et al. 2019). Additionally, non-aflatoxigenic *A. flavus* strains have been routinely applied on fields in Sub-Saharan Africa and the USA. In the 1980s, the non-aflatoxigenic *A. flavus* strain AF36 was isolated in Arizona (Cotty 1989). A point mutation in the polyketide synthase (*pks*A) gene of the AF gene cluster is responsible for coding a stop codon and thus leads to the inability to produce AF (Ehrlich and Cotty 2004). It was observed that AF36 had a good ability to compete against toxigenic *A. flavus* strains on cotton seeds (Cotty and Bhatnagar 1994). As the first commercially available biocontrol strain, in 2003 AF36 was registered by the US Environmental Protection Agency (EPA) as a biocontrol product for use on cotton fields in Arizona (EPA 2003). Later, it was also registered for the application on maize fields in Texas and Arizona (EPA 2011; Abbas et al. 2017). Strain AF36 clearly decreased the AF formation of aflatoxigenic *A. flavus* strains when it was used as a biocontrol agent (Cotty and Bhatnagar 1994). To date, the exact mechanism that is responsible for the reduced AF formation is not yet fully understood. Possible mechanisms are competition for nutrients and competitive exclusion, which is a thigmo-based response (Cotty and Bayman 1993; Huang et al. 2011). Thigmo-based responses are mediated by direct contact between the fungal cells and the environment (Almeida and Brand 2017).

To analyze the presence of naturally occurring non-aflatoxigenic *A. flavus* strains as well as those applied as biocontrol strains, methods are required that can distinguish and quantify aflatoxigenic and non-aflatoxigenic *A. flavus* strains in parallel. Several methods have been developed using the polymerase chain reaction (PCR), quantitative PCR (qPCR), or droplet digital PCR (ddPCR) (Criseo et al. 2001; Scherm et al. 2005; Degola et al. 2007; Latha et al. 2008; Abdel-Hadi et al. 2011; Jamali et al. 2013; Mahmoud 2015; Hua et al. 2018). However, these methods are only functional for non-aflatoxigenic *A. flavus* strains that have deletions at certain positions in the AF gene cluster. They are therefore not be able to identify *A. flavus* AF36 without these deletions as a non-aflatoxigenic strain.

Thus, the goal of this study was to design a ddPCR assay that simultaneously enables the specific detection and quantification of the non-aflatoxigenic *A. flavus* biocontrol strain AF36 as well as aflatoxigenic *A. flavus* strains. The developed ddPCR assay was subsequently used to analyze the competition and interaction of *A. flavus* AF36 and the aflatoxigenic *A. flavus* MRI19 co-inoculated on maize kernels and maize-based agar.

## Materials and methods

### Fungal strains and growth conditions

The following two *A. flavus* strains were used as competitors in this experiment: the aflatoxigenic *A. flavus* strain MRI19 of the culture collection of the Max Rubner-Institut, and the non-aflatoxigenic *A. flavus* strain AF36 (ATCC 96045, NRRL 18543) of the American Type Culture Collection. The *A. flavus* strain MRI19 represented a model strain for a moderate toxin-producing *A. flavus* strain from the environment. After its isolation from food, it was stored at −80 °C. For strain maintenance and the generation of spores, the strains were grown on MG agar (malt extract 17 g/L [Carl Roth, Karlsruhe, Germany], glucose 5 g/L [Carl Roth, Karlsruhe, Germany], agar [Agar–Agar Kobe I; Carl Roth, Karlsruhe, Germany] 16 g/L) for 7 days.

A spore suspension of each fungus containing 1.0*10^4^ spores per mL was prepared using Tween-80/NaCl-mixture (NaCl [Carl Roth, Karlsruhe, Germany] 9 g/L, Tween-80 [Serva, Heidelberg, Germany] 1 g/L, agar 1 g/L) (Schmidt-Heydt et al. 2012). For this, spores of initial spore suspensions were counted under a microscope (2 independent subsamples per strain) using a Thoma cell counting chamber (Paul Marienfeld GmbH & Co. KG, Lauda-Königshofen, Germany). The spore suspensions were subsequently diluted with Tween-80/NaCl to obtain a final concentration of 1.0*10^4^ spores per mL, which was verified by repeating the spore counting. Tween-80/NaCl was used, since the component polysorbate 80 acts as a nonionic surfactant and thus allows the homogeneous distribution of spores within the spore suspension. The spore suspensions of both strains were mixed to obtain the following ratios of the aflatoxigenic to the non-aflatoxigenic *A. flavus* strain in percent: 100:0, 90:10, 80:20, 70:30, 60:40, 50:50, 40:60, 30:70, 20:80, 10:90, and 0:100.

Maize kernels from a local store were used as a growth substrate. Twenty grams of maize per petri dish (Ø = 9 cm) was moisturized the day before inoculation by adding 3 mL of sterile water and mixing carefully. Three milliliters of each spore suspension mixture was pipetted onto the maize kernels, which were then mixed thoroughly. Further, maize-based agar plates (grounded maize kernels 50 g/L, agar [Agar–Agar Kobe I, Carl Roth, Karlsruhe, Germany] 12 g/L) were used, which were inoculated with three spots of 50 µL of the above-mentioned spore suspensions. For the extraction of the DNA, the maize-based agar plates were covered with a sterile cellophane sheet before inoculation. *A. flavus* was incubated on maize-based agar as well as on maize kernels at 25 °C in the dark. The experiments were repeated with fewer time points to verify the tendency of the results.

### DNA extraction

For the extraction of the genomic DNA of *A. flavus* grown on maize, three evenly overgrown maize kernels were picked out of each petri dish and were pooled. The DNA extraction was performed using the DNeasy Plant Mini Kit (Qiagen, Hilden, Germany) following the official protocol of the manufacturer with some modifications (Qiagen 2020). For the disruption and homogenization (2 × 30 s, 6.5 m/s), ceramic bead tubes (Type A, Macherey–Nagel, Düren, Germany) and a high-speed benchtop homogenizer (FastPrep-24™, MP Biochemicals Germany GmbH, Eschwege, Germany) were used. The amounts of buffer AP1, RNase A, and buffer P3 were increased to 850 µL, 8.5 µL, and 275 µL, respectively. For the DNA extraction of *A. flavus* grown on maize-based agar plates, 100 mg of mycelium was scraped off the cellophane sheet using a sterile scalpel after which the DNA extraction was performed in the same manner as described above. Only the amounts of buffer AP1, RNase A, and buffer P3 of the DNeasy Plant Mini Kit were adjusted to 600 µL, 6 µL, and 195 µL, respectively. The DNA concentration was measured with a fluorometer (Qubit 3; Thermo Fisher Scientific, Waltham, USA) and the quality of the DNA was verified using a NanoDrop 1000 Spectrophotometer (Thermo Fisher Scientific GmbH, Bremen, Germany).

### Design of the ddPCR assay

A single nucleotide polymorphism (G substituted by A) in the *pks*A gene of the AF gene cluster is responsible for the inability of *A. flavus* AF36 to produce AF (Ehrlich and Cotty 2004). This polymorphism was thus selected for the differentiation of the two *A. flavus* strains of interest. Primers and probes were designed by the Droplet Digital™ PCR Assays design software of Bio-Rad (Feldkirchen, Germany). The ID of the resulting assay is dMDS741862930 (Bio-Rad, Feldkirchen, Germany). The assay contains a primer pair that binds to the genomic DNA of both strains and generates the following 64 bp long amplicon: CGA GGT GAC CCT TGG TCT ACC ATT GTT TG[G/A] GGT CTG GAT CCC CAG CAA GCT CGT GAT CAG ATT G. Within this fragment, two TaqMan probes bind specifically to one of the two *A. flavus* strains. The probe specifically designed for *A. flavus* AF36 was labeled with the reporter dye 6-carboxyfluorescein (FAM), whereas the one specifically designed for *A. flavus* MRI19 was labeled with the reporter dye 5-hexachlorofluorescein (HEX) (Bio-Rad, Feldkirchen, Germany). Additionally, Iowa Black quencher (Bio-Rad, Feldkirchen, Germany) was utilized.

### ddPCR analysis

The ddPCR is a type of PCR that is characterized by a division of the reaction mix, including the target DNA, into ≤ 20,000 droplets per sample. These droplets are formed in a water–oil emulsion to separate DNA molecules, whereby each droplet can be regarded as an individual test tube. Classical end-point PCR is performed within these droplets. Based on their fluorescence amplitude, the droplets are subsequently assigned as positive (successful PCR amplification of the target sequence) or negative. The software uses the number of positive and negative droplets to calculate the concentration of the target gene in the analyzed sample (Hindson et al. 2011).

The ddPCR was performed by applying a QX200™ Droplet Digital PCR System (Bio-Rad, Feldkirchen, Germany). The reaction mix was composed of 10 µL of 2 × ddPCR Supermix for probes (no dUTP), 1 µL of 20 × primer/probe mix (900 nmol/L primers, 250 nmol/L probes), 1 µL of restriction enzyme mix (0.5 µL of Hae III 3000 units [New England BioLabs GmbH, Frankfurt, Germany], 0.4 µL of distilled water, 0.1 µL of buffer), 6 µL of distilled water, and 2 µL of fungal genomic DNA (1.25 ng/µL). In each run, an additional non-template control was included, in which water was used instead of DNA. The reaction mix was pipetted into a DG8™ Cartridge, and 70 µL Droplet Generation Oil for Probes was added for each sample. Droplets were generated by the QX200™ Droplet Generator. The droplet suspension was transferred into a 96-well plate (2 replicates per sample), which was sealed by the PX1™ PCR Plate Sealer. PCR amplification was performed by a PCR cycler (C1000 Touch Thermal Cycler; Bio-Rad, Feldkirchen, Germany) using the following cycling program: 95 °C for 10 min; 40 cycles of 94 °C for 30 s, 53 °C for 1 min; and 98 °C for 10 min. All steps were performed with a ramp speed of 2 °C/s. After the PCR, the amplification in the droplets was assessed by the QX200™ Droplet Reader based on the fluorescence signal. The resulting data was then analyzed using the software QuantaSoft™ version 1.7.4 and Quanta Soft™ Analysis Pro version 1.0 (Hindson et al. 2011; Pinheiro et al. 2012).

### High-performance liquid chromatography (HPLC)

For the determination of the AFB_1_ formation, four evenly overgrown maize kernels were transferred from each petri dish into a 2 mL micro-reaction tube. Of the maize-based agar plates, two agar plugs (Ø = 8 mm), one from the center and one from the edge of the colony, were taken by using a sterile corer and were also transferred into a 2 mL micro-reaction tube. Samples of maize kernels were analyzed in quintuplicate and of maize-based agar in triplicate. To extract mycotoxins, 1 mL of chloroform was added, and the maize kernels/agar plugs were shaken on a rotary shaker for 30 min at room temperature. The kernels/agar plugs were discarded, and the chloroform extract was filtered through a 0.2 µm PTFE membrane filter (Whatman™; Merck, Darmstadt, Germany) to eliminate the spores. Subsequently, 0.5 mL of the chloroform extract was evaporated to dryness in a vacuum concentrator (Savant; Thermo Fisher Scientific, Waltham, USA). For the quantification by HPLC, the extracts were re-dissolved in 100 µL of methanol before the extracts were again filtered through a 0.2 µm Phenex™-PTFE membrane filter (Phenomenex, Aschaffenburg, Germany) to eliminate residual impurities.

HPLC analysis was performed following an application note of Agilent Technologies (Barbas et al. 2005). Separation was carried out on a Hitachi Chromaster HPLC system (VWR International GmbH, Darmstadt, Germany) equipped with a ZORBAX Eclipse XDB C_18_ column (150 mm × 4.6 mm, 5 µm particle size; Agilent Technologies, Santa Carla, USA) at a flow rate of 0.8 mL per minute. The column oven was set to 40 °C and the injection volume was 10 µL. Analysis was performed under isocratic conditions using acetonitrile/methanol/water (10:40:50 [v:v:v]) as the mobile phase. AFB_1_ was quantified at 365 nm wavelength. AFB_1_ dissolved in acetonitrile (> 99%; Merck, Darmstadt, Germany) was used as a standard. The limit of quantification on the column for AFB_1_ was 0.05 µg/mL.

For the determination of the recovery of AFB_1_ after the extraction, maize-based agar and maize kernels were spiked with AFB_1_ before (pre-extract samples) and after (post-extract samples) the extraction process. The mean AFB_1_ concentration in the pre-extract samples was subsequently divided by the mean concentration in the post-extract samples. For the pre-extract samples (*n* = 5 per growth medium), maize-based agar plates (2 agar plugs per sample) and maize kernels (4 moistened maize kernels per sample) were spiked with AFB_1_ standard. Then, the toxin extraction was performed as described above. For post-extract samples (*n* = 5 per growth medium), toxin extracts from maize-based agar as well as maize kernels were spiked with AFB_1_ directly before HPLC analysis. The target AFB_1_ concentration of all injected spike samples was 1 µg/mL. The resulting data was analyzed using the software EZChrom Elite version 3.3.2 SP2 (Agilent Technologies, Santa Carla, USA). In addition to AFB_1_, *A. flavus* MRI19 produces aflatoxin B_2_. However, the amounts produced are very low in comparison to AFB_1_ (1–2%) and were thus not included in this study.

### Statistical analysis

Statistical analyses were performed with SPSS Statistics 26 (IBM, Armonk, NY, USA). The results were tested for normal distribution using the Shapiro–Wilk test and for homogeneity of variance using Levene’s test. Without fulfilling these requirements for parametric tests, the Mann–Whitney *U* test at a 95% confidence level (*p* value ≤ 0.05) was performed to compare the AF biosynthesis of the samples initially inoculated with 100% spores of the aflatoxigenic *A. flavus* to all other spore ratios of aflatoxigenic to non-aflatoxigenic *A. flavus*.

## Results

### Specificity, precision, and linearity of the ddPCR assay

**Fig. 1:**
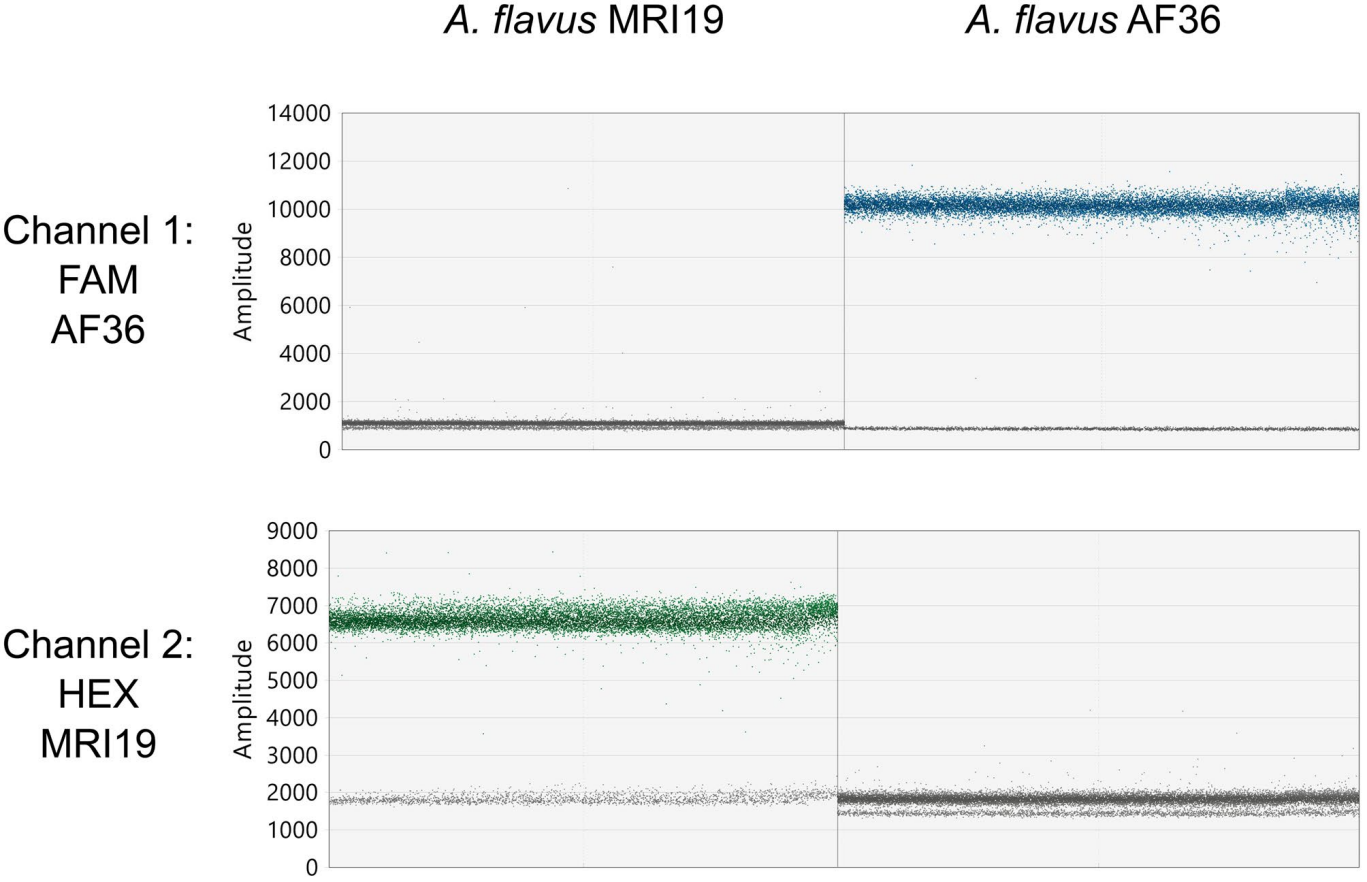
QuantaSoft™ Analysis Pro software display showing the specificity test of the ddPCR assay designed for the differentiation between the aflatoxigenic *A. flavus* MRI19 and the non-aflatoxigenic *A. flavus* AF36. The ddPCR was performed using pure DNA (2.5 ng) of only *A. flavus* MRI19 or AF36. Successful PCR amplification of the target gene (*pks*A) in individual droplets was detected by the binding of two differently labeled probes. A high amplitude (> 4,000) of a droplet detected by the QX200™ Droplet Reader indicated a successful amplification. The fluorophore FAM labeled the probe that bound to the DNA of strain AF36 and was detected in channel 1. The fluorophore HEX labeled the probe that bound to the DNA of strain MRI19 and was detected in channel 2. On the left side of the software display, the 1D-amplitude graph of a ddPCR reaction with DNA of *A. flavus* MRI19 is shown. Successful PCR amplification (shown by positive droplets at an amplitude of 5,000–8,000) was detected in channel 2 but not in channel 1. On the right side, the 1D-amplitude graph of a reaction with DNA of *A. flavus* AF36 is shown. Successful PCR amplification (shown by positive droplets at an amplitude of 8,000–12,000) was detected in channel 1 but not in channel 2

The specificity, measurement precision, and linearity of the ddPCR assay were checked to ensure the reliability of the assay. The specificity was verified by performing two ddPCR reactions with either 2.5 ng of DNA of the aflatoxigenic *A. flavus* strain MRI19 or the non-aflatoxigenic strain AF36 but including both probes (specific for either MRI19 or AF36) in each reaction (3 replicates per strain). The probe specific for *A. flavus* MRI19 was labeled with the fluorophore HEX, whereas the probe for *A. flavus* AF36 was labeled with the fluorophore FAM. No droplets with the opposite label were detected in channel 1 (detecting FAM, Fig. 1: upper row, left sample) of the QX200™ Droplet Reader in samples containing DNA of MRI19 or in channel 2 (detecting HEX, Fig. 1: lower row, right sample) in samples containing DNA of AF36. This verifies the high specificity of the two probes.

**Fig. 2:**
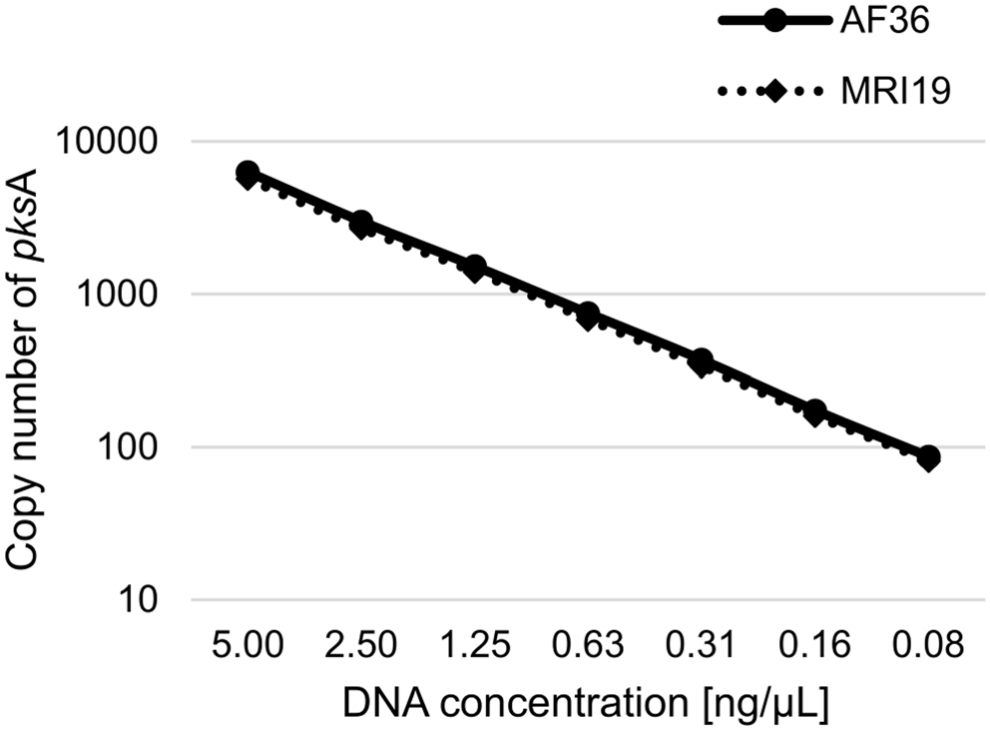
Line diagram showing the linearity test of the ddPCR assay designed for the differentiation between the aflatoxigenic *A. flavus* MRI19 and the non-aflatoxigenic *A. flavus* AF36. Pure DNA samples of *A. flavus* AF36 and *A. flavus* MRI19 were mixed 1:1 according to their concentrations measured using a fluorometer. A dilution series was prepared of this DNA mixture to obtain the final DNA concentrations of 5.00, 2.50, 1.25, 0.63, 0.31, 0.16, and 0.08 ng/µL (x-axis). The ddPCR was performed using 2 µL of these DNA solutions per 20 µL reaction mix (*n* = 4, per DNA concentration). The logarithmic y-axis shows the number of copies of the amplified fragment of the *pks*A gene of *A. flavus* MRI19 and *A. flavus* AF36 per 1 µL reaction mix

For the determination of the measurement precision and the linearity of the assay, pure DNA of *A. flavus* MRI19 and AF36 was mixed in a ratio of 1:1. The DNA concentrations were determined using a fluorometer (Qubit 3; Thermo Fisher Scientific, Waltham, USA). This DNA mixture was twofold serially diluted (5.00–0.08 ng/µL) and 4 replicates of each dilution were subjected to ddPCR. The reaction mixtures included the primers/probes of both fungal strains. The result of this experiment demonstrates the high linearity of the ddPCR assay (Fig. 2). A high linearity was further shown by coefficients of determination (*R*^2^) of 0.99938 and 0.99940 for the primers and probes of *A. flavus* MRI19 and *A. flavus* AF36.

Further, one concentration (1.25 ng/µL) of this DNA mixture was repeatedly measured eightfold for defining the measurement precision. For AF36 and MRI19, the arithmetic mean values and coefficients of variation were 1,520 and 1,381 copies/µL reaction mix as well as 1.89% and 0.87%, respectively. Furthermore, pure DNA of MRI19 and AF36 was mixed in the proportions 40:60 and 60:40 (according to the DNA concentrations determined using a fluorometer) to check the exactness of the determination of the ratios of both strains by ddPCR. The following mean ratios (and coefficients of variation) were obtained: 41.2% (2.9%):58.8% (6.8%) and 61.3% (0.6%):38.7% (0.5%) for MRI19:AF36, respectively.

### Recovery of the extraction process and precision and linearity of the HPLC method

The recovery of AFB_1_ after the extraction process was determined in a spiking experiment. The mean AFB_1_ concentration of samples spiked before extraction (*n* = 5) was divided by that of samples spiked after extraction (*n* = 5). For maize kernels, the recovery was 101.3% and for maize-based agar 80.3%. For the determination of the measurement precision of the HPLC analysis, one sample containing AFB_1_ (2.69 µg/mL) was tenfold measured. The coefficient of variation was 1.69%. The linearity was determined by measuring a dilution series of AFB_1_ standard with 6 different concentrations (2.01–0.13 µg/mL) in 2 replicates, respectively. The coefficient of determination (*R*^2^) was 0.99998.

### Competitiveness of AF-producing and non-producing strains of *A. flavus* on maize-based medium and maize kernels and its influence on the inhibition of the AF formation

**Fig. 3:**
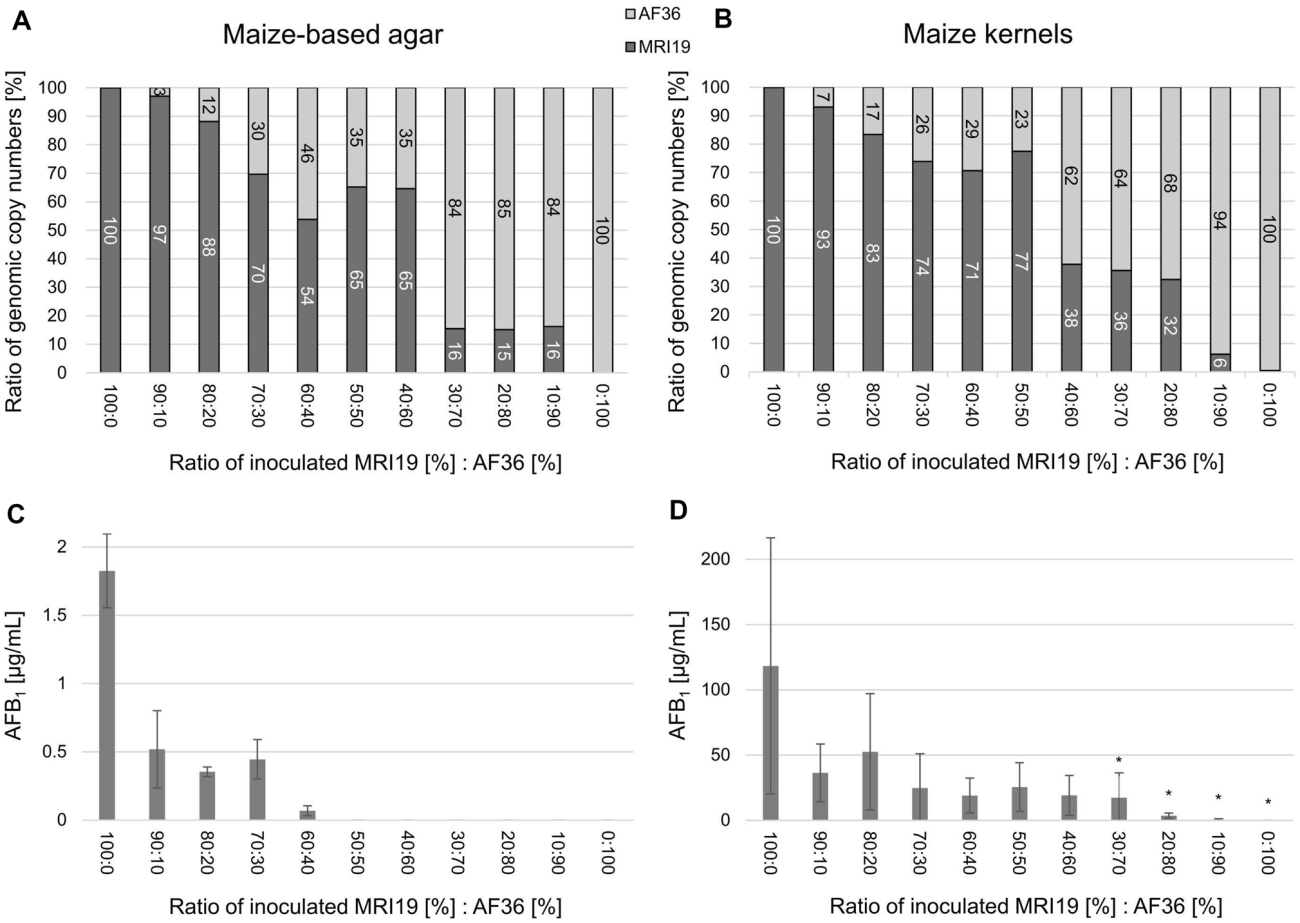
Ratios of genomic copy numbers of *A. flavus* MRI19 and *A. flavus* AF36 (**A**, **B**) and AFB_1_ biosynthesis of *A. flavus* MRI19 (**C**, **D**) on maize-based agar and maize kernels. Maize-based agar plates and maize kernels were inoculated with spore suspensions containing varying ratios of spores of *A. flavus* MRI19 to *A. flavus* AF36 (x-axes) and were incubated for 7 days at 25 °C. Fungal DNA was extracted and the final genomic equivalent ratios of the two strains were subsequently measured by ddPCR (pool of 3 samples, 2 technical replicates) using an assay designed for the differentiation of *A. flavus* MRI19 and AF36 (**A**, **B**). The final genomic equivalent ratios of *A. flavus* MRI19 are shown as dark columns, whereas those of *A. flavus* AF36 are shown as light columns. Further, the AFB_1_ formation of the aflatoxigenic *A. flavus* MRI19 at the different initially inoculated spore ratios was measured by HPLC (**C**, **D**) (per inoculation condition: *n* = 3 on maize-based agar, *n* = 5 on maize kernels). Asterisks indicate that the AFB_1_ biosynthesis of an inoculation condition is statistically significantly different (*p* value ≤ 0.05) to that of 100% initially inoculated spores of *A. flavus* MRI19

The interaction between the non-aflatoxigenic *A. flavus* strain AF36 and the aflatoxigenic *A. flavus* strain MRI19 on maize-based agar plates and on maize kernels was analyzed. Spore suspensions of the two strains were mixed in different ratios (MRI19 [%]:AF36 [%] such as 100:0, 90:10, 80:20, 70:30, 60:40, 50:50, 40:60, 30:70, 20:80, 10:90, 0:100). Spore mixtures were used for inoculating both growth media, which were then incubated for 7 days to get a first impression of the interaction. Afterward, the fungal DNA was isolated and the genome copy numbers of both strains were determined by ddPCR. A roughly comparable growth inhibition of *A. flavus* MRI19 (determined by its genomic equivalents) at an increasing spore input of *A. flavus* AF36 was observed for both growth substrates (Fig. 3A, B). However, growth inhibition of *A. flavus* MRI19 was rather irregular compared to the regular increase in the initial spore concentrations of *A. flavus* AF36, which indicates different levels of interactions at certain ranges of spore ratios. Within the different inoculation conditions of MRI19:AF36, the percentage of detected genomic equivalents of MRI19 was generally higher than its percentage in the initially inoculated spore suspensions, which indicates a growth advantage of MRI19 over AF36.

Additionally, the formation of AFs by *A. flavus* MRI19 was measured at the same inoculation conditions and at the same time as the genomic equivalents. The main metabolite was AFB_1_. Since aflatoxin B_2_ was measured to only approximately 1–2% of the levels of AFB_1_, it was not further investigated in this study. Aflatoxin G_1_ and G_2_ were not detected in the samples.

The non-aflatoxigenic *A. flavus* AF36 had a remarkable inhibiting effect on the AF formation of the aflatoxigenic strain *A. flavus* MRI19. On both maize-based agar and maize kernels, a clear inhibition of the AFB_1_ formation was already observed at an input spore ratio of MRI19 [%]:AF36 [%] of 90:10 compared to 100:0 (Fig. 3C, D). On maize-based agar plates, no AFB_1_ could be detected when ≥ 50% of the initially inoculated spores were of AF36. In contrast, on maize kernels > 90% of initial AF36 spores were necessary to reduce the AFB_1_ level to non-detectable levels.

### Competitiveness of AF-producing and non-producing strains of *A. flavus* on maize kernels over time and its influence on the inhibition of the AF formation

**Fig. 4:**
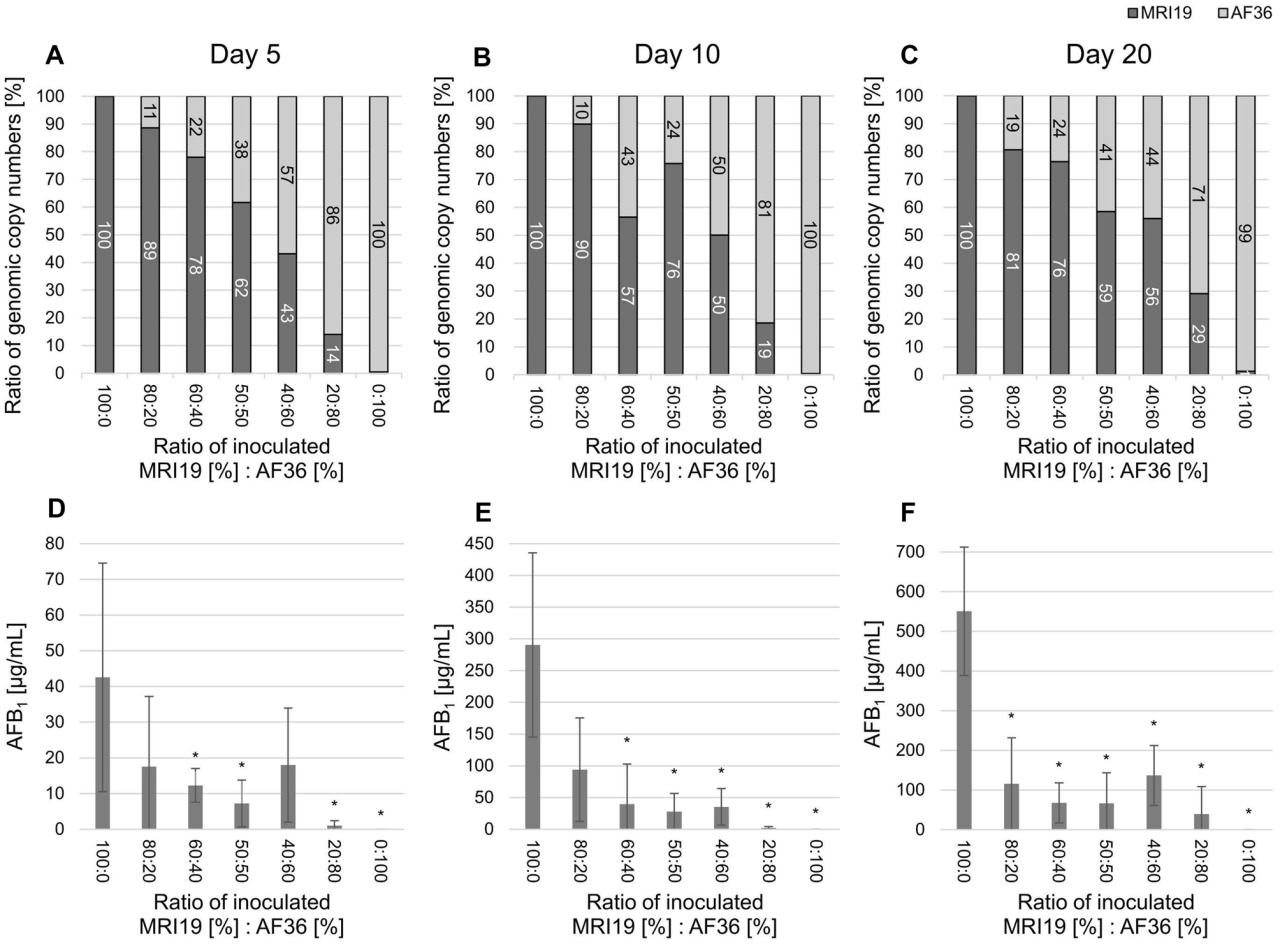
Ratios of genomic copy numbers of *A. flavus* MRI19 and *A. flavus* AF36 (**A**, **B**, **C**) and AFB_1_ biosynthesis of *A. flavus* MRI19 (**D**, **E**, **F**) on maize kernels. Maize kernels were inoculated with spore suspensions containing varying ratios of spores of *A. flavus* MRI19 and AF36 (x-axes) and were incubated for up to 20 days. The final genomic equivalent ratios of both *A. flavus* strains were determined after 5 (**A**), 10 (**B**), and 20 (**C**) days of incubation by ddPCR (pool of 3 samples, 2 technical replicates). The final genomic equivalent ratios of *A. flavus* MRI19 are shown as dark columns, whereas those of *A. flavus* AF36 are shown as light columns. The AFB_1_ formation of the aflatoxigenic *A. flavus* MRI19 was measured on day 5 (**D**), 10 (**E**), and 20 (**F**) of incubation by HPLC (per inoculation condition: *n* = 5). Asterisks show a statistically significant difference (*p* value ≤ 0.05) of the AFB_1_ biosynthesis of an inoculation condition compared to that of 100% initially inoculated spores of *A. flavus* MRI19

After the first experiment, in which the samples were incubated for 7 days, it was analyzed, whether the ratio of *A. flavus* MRI19 to *A. flavus* AF36 remained constant over a longer incubation period or whether one strain became dominant. Additionally, the AF formation under the various competing conditions was followed over time. Again, maize kernels were inoculated with spores of both strains in varying ratios (MRI19 [%]:AF36 [%] such as 100:0, 80:20, 60:40, 50:50, 40:60, 20:80, 0:100) and were incubated for up to 20 days. After 5, 10, and 20 days, samples were withdrawn to determine the actual genomic equivalents of both strains (Fig. 4A−C) and to measure the AF formation (Fig. 4D−F). A subtle but nevertheless visible change in the interaction behavior became obvious between the different sampling times. After an incubation of 5 days (Fig. 4A), genomic equivalents of *A. flavus* MRI19 decreased continuously with increasing amounts of initially inoculated AF36 spores. However, at inoculation conditions with ≥ 40% of MRI19 spores (MRI19 [%]:AF36 [%] such as 80:20, 60:40, 50:50), genomic equivalents of MRI19 were measured at a higher percentage than its spores were initially inoculated. This was observed for all three sampling times, except for the ratio 60:40 (for MRI19 [%]:AF36 [%]) on day 10, which might be an outlier. Only at the input spore ratio of 20:80 (for MRI19 [%]:AF36 [%]), strain MRI19 was detected at a lower percentage than initially inoculated. However, looking at the time kinetics of this ratio from day 5 to day 20, an increase of the actual genomic equivalents of MRI19 (from 14 to 29%) at the expense of AF36 was observed.

At all sampling times, the biosynthesis of AFB_1_ of *A. flavus* MRI19 was already clearly inhibited on maize kernels initially inoculated with 20% spores of the non-aflatoxigenic strain AF36 compared to samples inoculated with 100% spores of the toxigenic *A. flavus* strain MRI19 (pure MRI19 culture). This inhibition was higher than the low spore input ratio of AF36 would suggest and it became even more pronounced with prolonged incubation time. A further increase in the initially inoculated spores of AF36 from 40 to 60% had hardly any further inhibiting effect on the AF formation. In pure MRI19 culture, the AF formation continuously and remarkedly increased from day 5 to day 20. In contrast, the AF levels measured on maize kernels, inoculated with different ratios of both strains, increased at a much slower rate than in pure MRI19 culture. However, complete inhibition of the AF biosynthesis was not achieved and even at a spore ratio of 20:80 (MRI19 [%]:AF36 [%]), low levels of AFB_1_ were detected.

## Discussion

To our knowledge, this is the first report of a method that enables the parallel differentiation and quantification of the non-aflatoxigenic biocontrol strain *A. flavus* AF36 and aflatoxigenic *A. flavus* strains by ddPCR. A specific mutation in the *pks*A gene of the non-functional AF gene cluster of AF36 was chosen as a binding site for its specific probe. The probe designed for aflatoxigenic *A. flavus* strains targets the sequence of the functional *pks*A gene and is thus also applicable to other aflatoxigenic strains, as was shown in various experiments (data not shown).

In a first experiment, the developed ddPCR assay was tested to analyze the competitiveness of strain AF36 and the aflatoxigenic *A. flavus* MRI19 co-inoculated in different ratios on either maize-based agar plates or maize kernels. Similar interactions between both strains, that are evidently not directly dependent on the initial ratio of the spores of the competing strains, could be identified on both media. Comparing the interaction of both strains on maize kernels over time revealed some differences between short and prolonged incubation. After 5 days of incubation, the interaction still seemed to depend on the initially inoculated spore ratios of both strains. However, with increasing incubation time, the interaction became more irregular and less dependent on the initial spore ratios, which is comparable to the results of the previous experiment (Fig. 3). This suggests active interaction and competition between both strains with increasing incubation time. However, the differences observed are slight and could merely describe the tendency of the interaction in this biological system. Further experiments have to verify these findings.

Regardless of the incubation time, the aflatoxigenic *A. flavus* strain dominated at most inoculated spore ratios on both growth substrates and thus seems to be the moderately more competitive strain. Only at the spore ratio of 20:80 (for MRI19 [%]:AF36 [%]), the biocontrol strain appears to have a growth advantage after 5 days of incubation. This seems to be overcome with increasing incubation time. Unexpectedly, the AFB_1_ formation of the aflatoxigenic strain was already remarkably inhibited by a low amount of initially inoculated spores of the non-aflatoxigenic *A. flavus*. This suggests that the inhibition of the AF biosynthesis does not depend directly on the actual inoculated ratio of the two strains. It still has to be clarified in further experiments whether this inhibition acts on levels beyond the thigmotropic or competitive exclusion principles (Cotty and Bayman 1993; Almeida and Brand 2017).

The slightly higher competitiveness of the aflatoxigenic *A. flavus* strain might partly be explained by the inoculation of both strains at the same time point. Cotty and Bhatnagar (1994) showed for example that the inoculation of cotton bolls with strain *A. flavus* AF36 1 day prior to that of aflatoxigenic *A. flavus* strains increased the effectiveness of inhibiting the AF biosynthesis up to 100% compared to the simultaneous inoculation of the strains (Cotty and Bhatnagar 1994). Moreover, various other aspects may have influenced the respective competition. For example, *A. flavus* AF36 was originally isolated from cotton (Cotty and Bhatnagar 1994), so it might have a different, more effective behavior on cotton than on maize.

*A. flavus* produces AF during pathogenic (in the field) and saprophytic (during storage) growth and adapts its physiology accordingly (Reverberi et al. 2013). Dorner and Cole (2002) showed a higher effectiveness of biocontrol strains being applied on the field than after harvest and directly before storage. The conditions analyzed here rather tend to mimic storage conditions than field conditions. The focus of the current study is the analysis of the influence of the ratios of both strains in competition and not on the comparison of field and storage conditions. However, the results described here indicate that low amounts of AF36 can already strongly reduce AF biosynthesis without showing a strong competition against the growth of the aflatoxigenic strain. Although these results cannot directly be conveyed to field conditions, they do however demonstrate the effectiveness of AF36 to reduce AF synthesis in the natural habitat. Further research is needed to derive recommendations concerning the optimal application rate of non-aflatoxigenic biocontrol strains in the field.

The ddPCR was used for this analysis since it is a very precise method, which is necessary as the differentiation of the two strains is based on only one different nucleotide. Further, this method is robust against impurities from food systems, which may inhibit usual PCR or qPCR systems, and enables simple absolute quantification (Hindson et al. 2011; Morisset et al. 2013). Hua et al. (2018) already described a ddPCR system to differentiate between aflatoxigenic and atoxigenic *A. flavus* strains. Their method targets extensive, common deletions at the beginning of the AF gene cluster, which defines another set of non-aflatoxigenic strains (Chang et al. 2005). When analyzing the inhibiting activity of non-aflatoxigenic *A. flavus* strains in soil, Hua et al. (2018) showed that their inhibition was specific and strain-dependent. This could explain the to the current study contrasting observation of Hruska et al. (2014) that AF36 was the predominant and more robust strain on the surface of maize kernels co-inoculated (1:1) with the AF-producing *A. flavus* AF13. Taken together, the results of the current study and of the studies of Hua et al. (2018) and Hruska et al. (2014) suggest that the interaction between biocontrol strains and aflatoxigenic strains seems to partly be strain-specific, which makes predictions concerning effectiveness more difficult. Thus, the inclusion of different aflatoxigenic *A. flavus* strains for further experiments is recommended.

In the present study, the interaction of the non-aflatoxigenic biocontrol strain *A. flavus* AF36 and an aflatoxigenic *A. flavus* strain was analyzed by ddPCR using a newly designed ddPCR assay. During the review process, a further method for the detection and quantification of AF36 using qPCR was published, demonstrating the importance of the method developed in the current publication (Garcia-Lopez et al. 2021).

## Abbreviations

AF: Aflatoxin
AFs: Aflatoxins
AFB_1_: Aflatoxin B_1_
ddPCR: Droplet digital polymerase chain reaction
